# Indifferent effect of nonsteroidal anti-inflammatory drugs (NSAIDs) combined with fluconazole against multidrug-resistant *Candida auris*

**DOI:** 10.18502/cmm.5.3.1743

**Published:** 2019-09

**Authors:** Fatemeh Ahangarkani, Sadegh Khodavaisy, Shahram Mahmoudi, Tahereh Shokohi, Mohammad Sadegh Rezai, Hamed Fakhim, Eric Dannaoui, Saharnaz Faraji, Anuradha Chowdhary, Jacques F. Meis, Hamid Badali

**Affiliations:** 1 Student Research Committee, Mazandaran University of Medical Sciences, Sari, Iran; 2 Invasive Fungi Research Center, Mazandaran University of Medical Sciences, Sari, Iran; 3 Zoonoses Research Center, Research Institute for Health Development, Kurdistan University of Medical Sciences, Sanandaj, Iran; 4 Department of Medical Parasitology and Mycology, School of Public Health, Tehran University of Medical Sciences, Tehran, Iran; 5 Students Scientific Research Center, Tehran University of Medical Sciences, Tehran, Iran; 6 Department of Medical Parasitology and Mycology, Faculty of Medicine, Urmia University of Medical Sciences, Urmia, Iran; 7 Department of Medical Mycology, School of Medicine, Mazandaran University of Medical Sciences, Sari, Iran; 8 Pediatric Infectious Diseases Research Center, Mazandaran University of Medical Sciences, Sari, Iran; 9 Université Paris-Descartes, Faculté de Médecine, APHP, Hôpital Européen Georges Pompidou, Unité de Parasitologie-Mycologie, Service de Microbiologie, Paris, France; 10 Department of Medical Mycology, Vallabhbhai Patel Chest Institute, University of Delhi, Delhi, India; 11 Department of Medical Microbiology and Infectious Diseases, Canisius-Wilhelmina Hospital (CWZ), Nijmegen, The Netherlands; 12 Center of Expertise in Mycology Radboudumc/CWZ, Nijmegen, The Netherlands; 13 Pharmaceutical Sciences Research Center, Mazandaran University of Medical Sciences, Sari, Iran

**Keywords:** Antifungal drugs, Candida auris, Multidrug-resistant, NSAIDs

## Abstract

**Background and Purpose:**

Emergence and development of antifungal drug resistance in *Candida* species constitute a serious concern. *Candida auris* as an emerging multidrug-resistant fungus is the most important public health threat with high levels of mortality and morbidity. Almost all *C. auris* isolates are resistant to fluconazole, and there have been reports of elevated minimum inhibitory concentrations (MICs) to amphotericin B and echinocandins. To overcome the growing challenge of antifungal resistance, a valuable alternative option would be the use of drug combination.

**Materials and Methods:**

The present study evaluated the *in vitro* combination of nonsteroidal anti-inflammatory drugs (NSAIDs), such as ibuprofen, diclofenac and aspirin with fluconazole against fluconazole-resistant *C. auris* in comparison to other fluconazole-resistant *Candida* species, including *C. albicans*, *C. glabrata*, *C. parapsilosis, C. tropicalis, *and *C. krusei* originating from patients with candidiasis.

**Results:**

The MIC ranges of fluconazole-ibuprofen and fluconazole-diclofenac decreased from 32-256 to 32-128 and 16-256 µg/ml, respectively and remained the same for fluconazole-aspirin against *C. auris*. However, the combination of fluconazole with ibuprofen resulted in a synergistic effect for 5 strains, including *C. albicans* (n=2), *C. tropicalis* (n=1), *C. glabrata* (n=1), and *C. krusei* (n=1), by decreasing the MIC of fluconazole by 2-3 log_2_ dilutions.

**Conclusion:**

Although the interaction of NSAIDs with fluconazole was not synergistic against fluconazole-resistant *C. auris *isolates, no antagonism was observed for any combinations. Therefore, combination with newer azole agents needs to be conducted.

## Introduction

Fluconazole-resistance has emerged among *Candida* species since the beginning of the fluconazole era as a result of the selective pressure caused by prophylaxis and therapeutic management [1]. Emergence and development of antifungal drug resistance in *Candida* species isolates have serious concerns in terms of therapeutic failures mainly related to echinocandins and azoles resistance [2]*. Candida auris* has become an emerging serious public health threat [3] since the recognition of the first case from Japan in 2009 [4]. 

During the last ten years, the number of reported cases has drastically increased, including six outbreaks during 2013 and 2017 [5-10]. *Candida auris* infection has recently been reported from all continents. However, sporadic cases of *C. auris* have been reported from Austria, Belgium, Malaysia, the Netherlands, Norway, Russia, Switzerland, United Arab Emirates, and Iran [11-12]. Challenges in laboratory detection, unique predisposition to cause nosocomial outbreaks, and multidrug-resistant phenotypes of *C. auris* are the rule [13]. As a consequence of its ability to overcome antifungal therapy, *C. auris* continuously increases its ecological niche and resistance to environmental conditions. Accordingly, the multidrug-resistant phenotypes of *C. auris* may be associated with hospital-acquired infections [14-15]. 

Although most *C. auris *isolates are resistant to fluconazole and elevated the minimum inhibitory concentrations (MICs) of amphotericin B and echinocandins have been reported, echinocandins have been the most active drugs up to now [16-18]. Therefore, to overcome the growing challenge of antifungal resistance, a valuable alternative option would be the use of drug combinations [19]. 

Successful combination therapy for the treatment of fungal infectious diseases can achieve broader antifungal coverage and potentially reduce acquired resistance. Nonsteroidal anti-inflammatory drugs (NSAIDs), i.e., ibuprofen, diclofenac, and aspirin, show antifungal activity against *Candida *species alone or in combination with antifungal agents [20-25]. Antifungal activity of NSAIDs is related to the inhibition of the cyclooxygenase enzyme (COX) that leads to decreased prostaglandin levels. It seems that the suppression of oxylipins, oxygenated fatty acid metabolites derived from arachidonic acid, by NSAIDs can reduce the hyphal formation of *C. albicans* [20, 25].

Although the potential antifungal activities of NSAIDs against *Candida* species, such as the changes in prostaglandin production, reduction of extracellular polysaccharide, decrease in hyphal and biofilm formations, which may provide evidence for a combination strategy against pathogenic yeast, are known [20-25], no study has investigated the combination of NSAIDs and fluconazole against multi drug resistant *C. auris*. Therefore, the current study evaluated the *in vitro* combination of NSAIDs (i.e., ibuprofen, diclofenac, and aspirin) with fluconazole against fluconazole-resistant *C. auris* in comparison to other fluconazole-resistant *Candida* species.

## Materials and Methods

***Strains and identification***

A set of 16 clinically important *Candida* species comprising, *C. auris *(n=6), *C. albicans* (n=2), *C. glabrata* (n=2), *C. parapsilosis* (n=2), *C. tropicalis* (n=2), and *C. krusei* (n=2) originating from patients with candidiasis, was used. None *Candida auris* strains were isolated from pediatric patients suffering from hematological malignancies under chemotherapy and/or radiotherapy. All tested isolates have been previously identified by both DNA sequencing of internal transcribed spacer (ITS-rDNA) regions and MALDI-TOF mass spectrometer assay (MALDI Biotyper OC version 3.1, Bruker Daltonics, Bremen, Germany) [26]. Isolates were sub-cultured on Sabouraud Dextrose Agar (SDA, Difco) at 30 °C to ensure purity and viability. 

***In vitro antifungal susceptibility testing***

Fluconazole MICs were determined according to the guidelines of the Clinical and Laboratory Standards Institute (CLSI) (Table 1) [27, 28]. *Candida albicans *(ATCC 64124) was used as a reference strain for azole resistance with mutation in the azole target *Erg11* [29].

**Table 1:** Minimum inhibitory concentrations of fluconazole and nonsteroidal anti-inflammatory drugs (i.e., ibuprofen, diclofenac, and aspirin) alone and in combination against *Candida auris* and other fluconazole-resistant *Candida *species

**No.**	**Strains**	**Country of origin**	**MIC values (µg/ml)**							
			**Alone**					**In combination (FICI/Interpretation)**		
			**FLZ**	**IBR**	**DIC**	**ASA**	**FLZ/IBR**		**FLZ/DIC**	**FLZ/ASA**
1	*C. auris*	India	128	4096	2048	2048	128/2048 (1.5/I)		128/2048 (2/I)	64/512 (0.75/I)
2	*C. auris*	India	256	2048	8192	2048	128/1024 (1/I)		64/2048 (0.5/I)	256/1024 (1.5/I)
3	*C. auris*	India	256	2048	2048	4096	64/2048 (1.25/I)		128/1024 (1/I)	256/1024 (1.25/I)
4	*C. auris*	India	256	4096	8192	2048	128/2048 (1/I)		256/2048 (1.25/I)	128/1024 (1/I)
5	*C. auris*	India	256	4096	2048	2048	128/1024 (0.75/I)		64/1024 (0.75/I)	128/512 (0.75/I)
6	*C. auris*	Iran	32	2048	4096	1024	32/2048 (2/I)		16/2048 (1/I)	32/1024 (2/I)
7	*C. albicans*	Iran	256	512	1024	1024	32/64 (0.25/S)		32/256 (0.375/S)	16/256 (0.3125/S)
8	*C. albicans*	Iran	256	1024	1024	1024	16/256 (0.3125/S)		128/1024 (1.5/I)	8/128 (0.1562/S)
9	*C. tropicalis*	Iran	64	256	2048	2048	16/32 (0.375/S)		64/1024 (1.5/I)	8/512 (0.375/S)
10	*C. tropicalis*	Iran	16	256	512	1024	64/256 (5/A)		64/512 (5/A)	16/512 (1.5/I)
11	*C. parapsilosis*	Iran	128	2048	2048	2048	64/1024 (1/I)		128/512 (1.25/I)	128/2048 (2/I)
12	*C. parapsilosis*	Iran	64	1024	1024	2048	64/512 (1.5/I)		64/1024 (2/I)	32/2048 (1.5/I)
13	*C. glabrata*	Iran	16	4096	2048	2048	16/1024 (1.25/I)		16/2048 (2/I)	16/2048 (2/I)
14	*C. glabrata*	Iran	256	2048	4096	2048	32/256 (0.25/S)		256/2048 (1.5/I)	128/1024 (1/I)
15	*C. krusei*	Iran	128	1024	2048	1024	16/64 (0.1875/S)		128/2048 (2/I)	128/2048 (3/I)
16	*C. krusei*	Iran	64	1024	2048	1024	32/512 (1/I)		64/2048 (2/I)	64/1024 (2/I)

MIC: Minimum inhibitory concentrations, FICI: Fractional inhibitory concentration index, FLZ: fluconazole, IBR: ibuprofen, DIC: diclofenac, ASA: aspirin, I: Indifference, S: Synergism, A: Antagonism

***In vitro combination testing***

A checkerboard microdilution assay based on the CLSI reference technique was performed in 96-well microtiter plates to evaluate the *in vitro* interactions between NSAIDs (i.e., ibuprofen, diclofenac, and aspirin) and fluconazole against azole-resistant *Candida* species [30]. The drugs were dissolved in 100% dimethyl sulfoxide (DMSO), and the concentrations were within the range of 0.5 to 128 µg/ml for fluconazole (FLZ; Pfizer, Groton, CT, USA), 32 to 2048 µg/ml for ibuprofen (IBR, Sigma), and aspirin (ASA, Sigma) and 64 to 4096 µg/ml for diclofenac (DIC, Sigma). Briefly, 50 µl of each concentration of fluconazole was dispensed into the columns of 1 to 10, and 50 µl of NSAIDs (IBR or DIC or ASA) was added to the rows of A to G of 96-well microplates. The H row and column 11 contained fluconazole and NSAIDs alone, respectively. In addition, column 12 was used as the drug-free growth control. The inoculum was prepared using fresh colonies, and their density was adjusted to 1-3 × 10^3^ CFU/ml at 530 nm wavelength to a percentage transmission within the range of 75-77% according to CLSI M27-S4 guide [26].

For each drug combination plate, 100 µl of inoculum was added to all the wells, and the plates were incubated at 35 °C for 24 h. The test was performed in duplicate on two separate days. The MICs were visually determined as the lowest concentration of drug that reduced growth (≥50% inhibition) in comparison to growth controls. For the determination of drug interactions, fractional inhibitory concentration index (FICI) was calculated as MIC drug A in combination/MIC drug A alone plus MIC drug B in combination/MIC drug B alone and interpreted as synergism (FICI≤0.5), indifference (0.5<FICI≤4), and antagonism (FICI>4) [30].

## Results

Table 1 summarizes the results of the MICs of fluconazole and NSAIDs (i.e., ibuprofen, diclofenac, and aspirin) alone and in combination. The MIC ranges of the individual tested NSAIDs against *C. auris *isolates were 256-4096, 512-8192, and 1024-4096 µg/ml for ibuprofen, diclofenac, and aspirin, respectively. However, the MIC range of fluconazole against *C. auris *was 32-256 µg/ml. Based on MIC values, all tested strains were fully resistant to fluconazole, except for one strain of *C. glabrata* that was susceptible dose-dependent, and the MIC range of fluconazole against other non-*albicans* species was 16-256 µg/ml. 

Checkerboard microdilution assays of *C. auris* showed that when fluconazole was combined with NSAIDs, the MIC ranges of fluconazole-ibuprofen and fluconazole-diclofenac decreased from 32-256 to 32-128 and 16-256 µg/ml, respectively. In addition, it remained the same for fluconazole-aspirin (Table 1) suggestive of the indifferent combinations between fluconazole with ibuprofen, diclofenac, or aspirin (FICI>0.5 to ≤4). 

The most common obtained result of the checkerboard assay in this study was indifferent (i.e., 62.5%, 87.5%, and 81.25% for fluconazole-ibuprofen, fluconazole-diclofenac, and fluconazole-aspirin, respectively). However, the *in vitro* combination of fluconazole with ibuprofen resulted in a synergistic effect for 5 strains, including *C. albicans* (n=2), *C. tropicalis* (n=1), *C. glabrata* (n=1), and *C. krusei* (n=1), by decreasing the MIC of fluconazole by 2-3 log_2_ dilutions. 

Synergistic interactions between fluconazole and aspirin were recorded for three strains and observed in only one instance synergism when fluconazole was combined with diclofenac. None of the analyzed data sets had FICI higher than 4, indicating that no antagonism was observed except for one *C. tropicalis* strain in which the combinations of fluconazole with ibuprofen and fluconazole with diclofenac showed an antagonistic effect.

## Discussion

*Candida auris *is one of the top-ten most serious fungal pathogens to date [31]. Despite the application of antifungal therapy, management is a big challenge because* C. auris *is a persistent colonizer and difficult to eradicate from the hospital environment [32, 33]. Moreover, the ability to form biofilms, adherence to catheter material, and resistance to the major antifungal drugs may have contributed to its persistence and nosocomial transmission [7, 10]. Pan-resistance of *C. auris* has been reported with the high MICs of fluconazole (90%), echinocandins (2%), amphotericin B (8%), and voriconazole (2.3%) [16]. 

In addition, Khan et al. reported that 100% (56/56) of the *C. auris* isolates were fluconazole-resistant 73% (41/56) and 23 % (13/56) of the isolates exhibited cross-resistance to voriconazole and amphotericin B, respectively. Furthermore, 20% (11/56) of the isolates were simultaneously resistant to fluconazole, voriconazole, as well as amphotericin B, and one isolate demonstrated resistance to caspofungin and micafungin [34]. 

Our previous experience on the interaction of caspofungin with voriconazole and micafungin with fluconazole against multidrug-resistant *C. auris* revealed indifferent activities against all strains (FICI, 0.62 to 2). Nonetheless, the synergistic effects of micafungin with voriconazole were observed (FICI, 0.15 to 0.5) and no antagonism was identified for any combinations [35]. 

In a study carried out by Eldesouky et al., it was reported that sulfamethoxazole had the highest synergistic activity with fluconazole against both *C. albicans* and *C. auris* [36]*.* In this regard, the fluconazole MICs of *C. auris* were reduced by 8 log_2 _dilutions resulting in an FICI of 0.156 [37].

In our previous study, geldanamycin interacted synergistically with both fluconazole and itraconazole against *C. albicans*, *C. glabrata*, and *C. parapsilosis*. However these combinations were indifferent against *C. auris* [38]. Significant inhibitory effects of NSAIDs on the growth of *Candida* species, *Trichosporon asahii,* and *Cryptococcus* species have been previously reported [20]. 

Furthermore, there have been reports of the *in vitro* synergistic effect of fluconazole with ibuprofen, sodium salicylate, or propylparaben against *C. albicans* [21, 23]. In a study carried out by Scott et al. a synergistic interaction was observed between NSAIDs and fluconazole against azole-resistant *C.* *albicans.* Moreover, NSAIDs were able to revert the azole resistance of *C. albicans *at clinically relevant concentrations, *in vitro* [23]. In this regard, the results of the aforementioned study are in line with the findings of the present study. 

In the present study, the combination of fluconazole with ibuprofen resulted in a synergistic effect against *C. albicans* (n=2), *C. tropicalis* (n=1), *C. glabrata* (n=1), and *C. krusei* (n=1). Similar to the findings of the present study, Pina-Vaz et al. demonstrated that the combination of ibuprofen with fluconazole had synergic activity in four fluconazole-resistant strains, including *C. glabrata* (n=2), C. *albicans *(n=1), and *C. krusei* (n=1). Furthermore, they recommended the practicability of using ibuprofen alone or in combination with azoles in the treatment of candidiasis, particularly when topically applied [21].

## Conclusion

In summary, the results of the present study showed that the interaction of NSAIDs and fluconazole had a synergistic activity against azole-resistant *C. albicans* and *C. tropicalis*, but indifferent effects were observed for both resistant *C. auris* and *C. krusei*. Although the interaction of NSAIDs and fluconazole was not synergistic against resistant *C. auris* isolates, the combination with newer azole agents would be another option. However, the significance of these *in vitro* findings remains elusive and requires consideration.

## References

[1] Lamoth F, Lockhart SR, Berkow EL, Calandra T (2018). Changes in the epidemiological landscape of invasive candidiasis. J Antimicrob Chemother..

[2] Cortegiani A, Misseri G, Chowdhary A (2019). What’s new on emerging resistant Candida species. Intensive Care Med.

[3] Meis JF, Chowdhary A (2018). Candida auris: a global fungal public health threat. Lancet Infect Dis.

[4] Satoh K, Makimura K, Hasumi Y, Nishiyama Y, Uchida K, Yamaguchi H (2009). Candida auris sp a novel ascomycetous yeast isolated from the external ear canal of an inpatient in a Japanese hospital. Microbiol Immunol.

[5] Calvo B, Melo AS, Perozo-Mena A, Hernandez M, Francisco EC, Hagen F (2016). First report of Candida auris in America: Clinical and microbiological aspects of 18 episodes of candidemia. J Infect.

[6] Ruiz-Gaitán A, Moret AM, Tasias-Pitarch M, Aleixandre-López AI, Martínez-Morel H, Calabuig E (2018). An outbreak due to Candida auris with prolonged colonisation and candidaemia in a tertiary care European hospital. Mycoses.

[7] Chow NA, Gade L, Tsay SV, Forsberg K, Greenko JA, Southwick KL (2018). Multiple introductions and subsequent transmission of multidrug-resistant Candida auris in the USA: a molecular epidemiological survey. Lancet Infect Dis.

[8] Araúz AB, Caceres DH, Santiago E, Armstrong P, Arosemena S, Ramos C (2018). Isolation of Candida auris from 9 patients in Central America: importance of accurate diagnosis and susceptibility testing. Mycoses..

[9] Schelenz S, Hagen F, Rhodes JL, Abdolrasouli A, Chowdhary A, Hall A (2016). First hospital outbreak of the globally emerging Candida auris in a European hospital. Antimicrob Resist Infect Control..

[10] Eyre DW, Sheppard AE, Madder H, Moir I, Moroney R, Quan TP (2018). A Candida auris outbreak and its control in an intensive care setting. N Engl J Med.

[11] Saris K, Meis JF, Voss A (2018). Candida auris. Curr Opin Infect Dis.

[12] Abastabar M, Haghani I, Ahangarkani F, Rezai MS, Taghizadeh Armaki M, Roodgari S (2019). Candida auris otomycosis in Iran and review of recent literature. Mycoses.

[13] Forsberg K, Woodworth K, Walters M, Berkow EL, Jackson B, Chiller T (2019). Candida auris: the recent emergence of a multidrug-resistant fungal pathogen. Med Mycol.

[14] Biswal M, Rudramurthy SM, Jain N, Shamanth AS, Sharma D, Jain K (2017). Controlling a possible outbreak of Candida auris infection: lessons learnt from multiple interventions. J Hosp Infect.

[15] Lee WG, Shin JH, Uh Y, Kang MG, Kim SH, Park KH (2011). First three reported cases of nosocomial fungemia caused by Candida auris. J Clin Microbiol.

[16] Chowdhary A, Prakash A, Sharma C, Kordalewska M, Kumar A, Sarma S (2018). A multicenter study of antifungal susceptibility patterns among 350 Candida auris isolates (2009-17) in India: role of the ERG11 and FKS1 genes in azole and echinocandin resistance. J Antimicrob Chemother.

[17] Escandón P, Chow NA, Caceres DH, Gade L, Berkow EL, Armstrong P (2019). Molecular epidemiology of Candida auris in Colombia reveals a highly related, countrywide colonization with regional patterns in Amphotericin B resistance. Clin Infect Dis.

[18] Mathur P, Hasan F, Singh PK, Malhotra R, Walia K, Chowdhary A (2018). Five-year profile of candidaemia at an Indian trauma centre: high rates of Candida auris blood stream infections. Mycoses.

[19] Butts A, Krysan DJ (2012). Antifungal drug discovery: something old and something new. PLoS Pathog.

[20] Lan YB, Huang YZ, Qu F, Li JQ, Ma LJ, Yan J (2017). Time course of global gene expression alterations in Candida albicans during infection of HeLa cells. Bosn J Basic Med Sci.

[21] Pina-Vaz C, Sansonetty F, Rodrigues AG, Martinez-De-Oliveira J, Fonseca AF, Mårdh PA (2000). Antifungal activity of ibuprofen alone and in combination with fluconazole against Candida species. J Med Microbiol.

[22] de Quadros AU, Bini D, Pereira PA, Moroni EG, Monteiro MC (2011). Antifungal activity of some cyclooxygenase inhibitors on Candida albicans: PGE2-dependent mechanism. Folia Microbiol.

[23] Scott EM, Tariq VN, McCrory RM (1995). Demonstration of synergy with fluconazole and either ibuprofen, sodium salicylate, or propylparaben against Candida albicansin vitro. Antimicrob Agents Chemother.

[24] Krol J, Nawrot U, Bartoszewicz M (2018). Anti-candidal activity of selected analgesic drugs used alone and in combination with fluconazole, itraconazole, voriconazole, posaconazole and isavuconazole. J Mycol Med.

[25] Yucesoy M, Oktem IM, Gulay Z (2000). In-vitro synergistic effect of fluconazole with nonsteroidal anti-inflammatory agents against Candida albicans strains. J Chemother.

[26] Aslani N, Janbabaei G, Abastabar M, Meis JF, Babaeian M, Khodavaisy S (2018). Identification of uncommon oral yeasts from cancer patients by MALDI-TOF mass spectrometry. BMC Infect Dis.

[27] CLSI (2008). Reference method for broth dilution antifungal susceptibility testing of yeast; approved standards, (M27-A3).

[28] Pfaller MA (2012). Antifungal drug resistance: mechanisms, epidemiology, and consequences for treatment. Am J Med.

[29] Liu JY, Shi C, Wang Y, Li WJ, Zhao Y, Xiang MJ (2015). Mechanisms of azole resistance in Candida albicans clinical isolates from Shanghai, China. Res Microbiol.

[30] Odds FC (2003). Synergy, antagonism, and what the chequerboard puts between them. J Antimicrob Chemother.

[31] Hyde KD, Al-Hatmi AM, Andersen B, Boekhout T, Buzina W, Dawson TL (2018). The world’s ten most feared fungi. Fungal Divers.

[32] Chowdhary A, Voss A, Meis JF (2016). Multidrug-resistant Candida auris: 'new kid on the block' in hospital-associated infections?. J Hosp Infect.

[33] Chow NA, de Groot T, Badali H, Abastabar M, Chiller TM, Meis JF (2019). Potential fifth clade of Candida auris, Iran, 2018. Emerg Infect Dis.

[34] Khan Z, Ahmad S, Al-Sweih N, Joseph L, Alfouzan W, Asadzadeh M (2018). Increasing prevalence, molecular characterization and antifungal drug susceptibility of serial Candida auris isolates in Kuwait. PLoS One.

[35] Fakhim H, Chowdhary A, Prakash A, Vaezi A, Dannaoui E, Meis JF (2017). In vitro interactions of echinocandins with triazoles against multidrug-resistant Candida auris. Antimicrob Agents Chemother.

[36] Eldesouky HE, Mayhoub A, Hazbun TR, Seleem MN (2018). Reversal of azole resistance in Candida albicans by sulfa antibacterial drugs. Antimicrob Agents Chemother.

[37] Eldesouky HE, Li X, Abutaleb NS, Mohammad H, Seleem MN (2018). Synergistic interactions of sulfamethoxazole and azole antifungal drugs against emerging multidrug-resistant Candida auris. Int J Antimicrob Agents.

[38] Mahmoudi S, Rezaie S, Ghazvini RD, Hashemi SJ, Badali H, Foroumadi A (2019). In Vitro interaction of geldanamycin with triazoles and echinocandins against common and emerging Candida species. Mycopathologia..

